# Association between triglyceride glucose–relative fat mass index and obstructive sleep apnea risk: A cross-sectional study from NHANES

**DOI:** 10.1097/MD.0000000000051331

**Published:** 2026-10-23

**Authors:** Yunzhou Zheng, Shanshan Zhang, Mingqiang Mi

**Affiliations:** a Clinical Laboratory, Bethune International Peace Hospital, Shijiazhuang, People’s Republic of China; b Department of Procurement, Bethune International Peace Hospital, Shijiazhuang, People’s Republic of China.

**Keywords:** cross-sectional study, National Health and Nutrition Examination Survey, obstructive sleep apnea, relative fat mass, triglyceride glucose index

## Abstract

The triglyceride glucose (TyG) index is correlated with insulin resistance and obstructive sleep apnea (OSA), whereas the relative fat mass (RFM) index is linked to obesity and OSA. However, the relationship between the triglyceride glucose–relative fat mass (TyG–RFM) index and OSA has been less studied in the US population. This research was designed to investigate the association between the TyG–RFM index and OSA prevalence. Data for this cross-sectional analysis were drawn from the 2005 to 2008 and 2015 to 2018 cycles of the National Health and Nutrition Examination Survey, including participants aged ≥20 years. Weighted multivariate logistic regression and restricted cubic splines were employed to examine the relationship between the TyG–RFM index and OSA. Stratified and sensitivity analyses were undertaken to test the consistency of the association. Of the 7283 participants, the mean age was 46.9 (standard deviation = 16.7) years, and 48.7% were male. Among them, 3616 individuals exhibited symptoms of OSA. The fitted curve demonstrated a linear positive relationship between the TyG–RFM index and OSA (*P* for nonlinearity = 0.292). In the fully adjusted model, each 10-unit increase in the TyG–RFM index was associated with a 4% higher OSA risk (odds ratio, 1.04; 95% confidence interval, 1.02–1.06). When analyzed by quintiles, participants in the highest quintile had 95% greater odds of OSA compared with those in the lowest quintile (odds ratio, 1.95; 95% confidence interval, 1.29–2.95). Stratified and sensitivity analyses confirmed the stability of the primary results. This cross-sectional research showed that the TyG–RFM index was associated with OSA risk among US adults. These results may aid in OSA risk stratification and early screening.

## 1. Introduction

Obstructive sleep apnea (OSA) is a common sleep disorder involving recurrent collapse of the upper airway during sleep, which leads to intermittent hypoxia and fragmentation of sleep architecture.^[1–3]^ Epidemiological reports suggest that the global OSA population approaches 1 billion individuals, approximately 80% of whom remain undiagnosed and untreated.^[4,5]^ Among these, nearly half are moderate-to-severe cases, with a higher prevalence observed in males and obese populations.^[6,7]^ OSA is linked to a heightened risk of multiple cardiometabolic conditions, such as hypertension, diabetes, atherosclerosis, and obesity-related metabolic dysregulation.^[8,9]^ Furthermore, OSA constitutes a significant risk factor for elevated all-cause and cardiovascular mortality.^[10]^ Consequently, discovering novel and precise biomarkers represents a pressing clinical need to enhance OSA risk assessment and facilitate early intervention.

The triglyceride glucose (TyG) index serves as a valid surrogate marker of insulin resistance and is used to predict diabetes and OSA.^[11]^ Relative fat mass (RFM), which incorporates sex, height, and waist circumference, offers a more accurate estimate of body fat percentage across diverse populations compared with body mass index (BMI).^[12]^ Previous research has reported associations between TyG or composite indicators combining TyG and measures of body fat distribution (such as waist circumference, BMI) with the risk and severity of OSA, suggesting that integrated markers of metabolism and adiposity may be useful for OSA risk stratification beyond conventional measurements.^[13,14]^ BMI poorly distinguishes fat distribution and metabolic risk, and single indicators may not fully capture the intersecting contributions of metabolic dysregulation and adiposity to OSA pathogenesis.^[12]^ The product of TyG and RFM (TyG–RFM) integrates 2 metrics reflecting insulin resistance and body fat distribution into a single indicator, thereby overcoming the limitations of relying on a single measure. However, the association between TyG–RFM and OSA risk has not yet been evaluated in a US population.

Using large-scale data from the National Health and Nutrition Examination Survey (NHANES), this study examined the association between the TyG–RFM index and OSA, hypothesizing that a higher TyG–RFM index would correlate with greater OSA risk. Our findings could offer a novel, biomarker-based strategy for OSA risk stratification and early screening.

## 2. Methods

### 2.1. Data sources

The data utilized in this research were obtained from the publicly accessible NHANES database (http://www.cdc.gov/nchs/nhanes; accessed November 10, 2025). NHANES is an annual survey conducted by the National Center for Health Statistics to examine the health and nutritional status of a nationally representative, cross-sectional sample of the US population across all age groups. This analysis included data from four 2-year survey cycles (2005–2008 and 2015–2018). The study protocol received approval from the Ethics Review Board of the National Center for Health Statistics in compliance with applicable ethical regulations. All participants provided written informed consent before enrollment. Analysis of these deidentified, publicly available data adhered to the Strengthening the Reporting of Observational Studies in Epidemiology guidelines. Given the public and anonymized nature of the data, no further ethical clearance was necessary for this investigation.

### 2.2. Study design and participants

This cross-sectional study enrolled 39,722 participants spanning 4 NHANES survey cycles (2005–2006, 2007–2008, 2015–2016, and 2017–2018). After excluding individuals younger than 20 years (n = 17,520), those without OSA-related data (n = 8), and participants with a missing TyG–RFM index (n = 13,224), we further excluded individuals with incomplete covariate data or missing survey weights (n = 1687). Ultimately, 7283 individuals were included in the analytic sample (Fig. 1).

**Figure 1.**
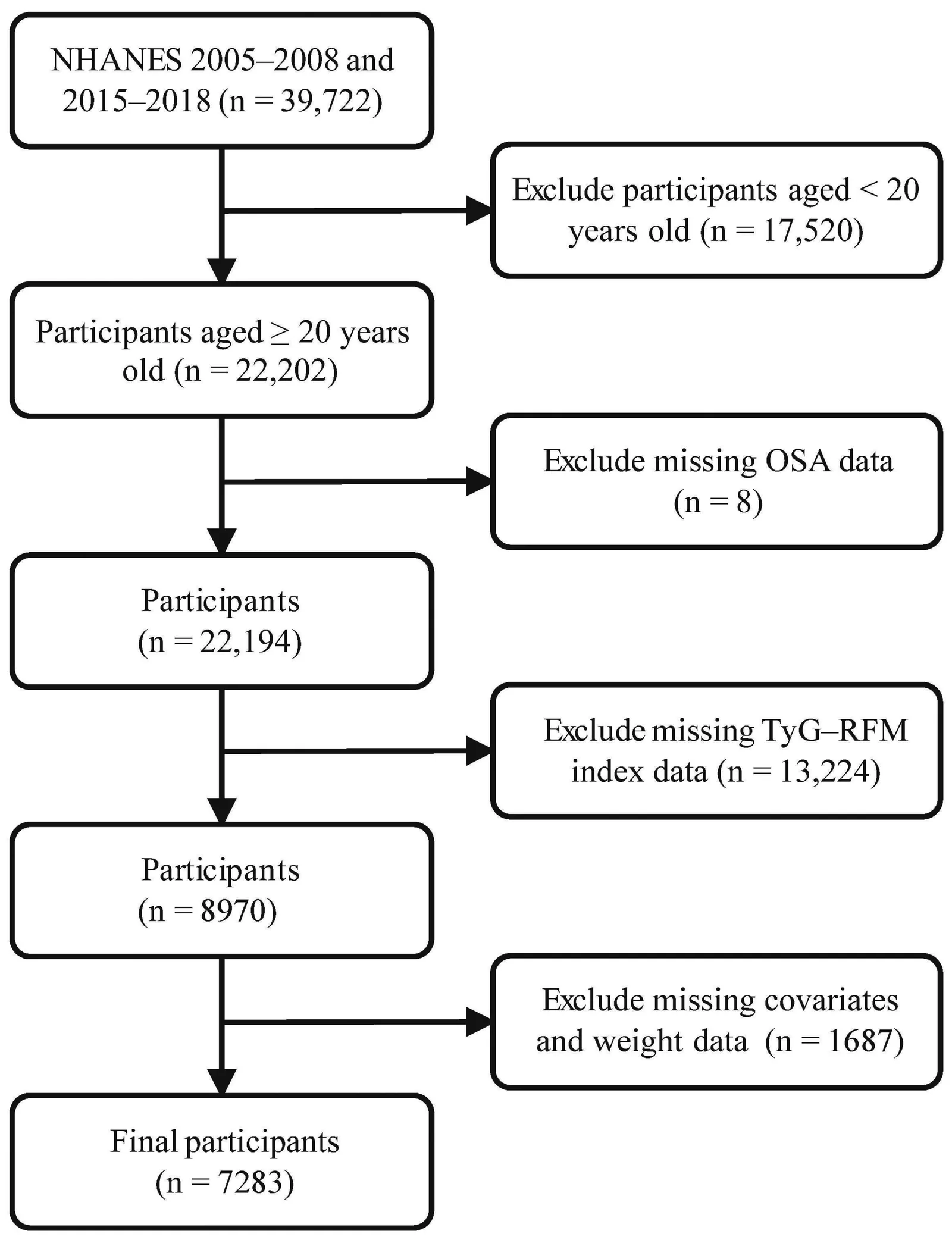
Flowchart of the study design. NHANES = National Health and Nutrition Examination Survey, OSA = obstructive sleep apnea, TyG–RFM = triglyceride glucose–relative fat mass.

### 2.3. Definition of TyG–RFM index

The TyG–RFM index was defined by the formula^[12,13]^:

TyG–RFM index = TyG–RFM × RFM

TyG = Ln (fasting triglyceride [mg/dL] × fasting blood glucose [mg/dL]/2)

RFM (women) = 76 − (20 × height [cm]/waist circumference [cm])

RFM (men) = 64 − (20 × height [cm]/waist circumference [cm])

### 2.4. Definition of OSA

OSA was defined using participant responses to 3 key survey questions^[15]^: “In the past year, have you snored at least 3 times per week?”; “In the past year, did you experience wheezing, snoring, or breathing pauses 3 or more times per week?”; and “In the past year, even with 7 or more hours of sleep on weekdays, did you feel excessively sleepy during the day for 16 to 30 days per month?” A positive response to any of these items indicated the presence of OSA-related symptoms. It should be noted that the OSA diagnosis in this study was based on self-reported symptoms rather than clinical assessment.

### 2.5. Covariates

Based on previous literature^[3,16,17]^ and clinical experience, the included covariates comprised age, sex, race/ethnicity, educational attainment, marital status, poverty income ratio, BMI, smoking status, alcohol consumption, hypertension, coronary heart disease, diabetes, physical activity, and sleep duration. Race/ethnicity, marital status, diabetes, coronary heart disease status, and educational attainment were self-reported by participants.^[18,19]^ Smoking status was classified into 3 categories: never smokers (those who had smoked fewer than 100 cigarettes in their lifetime), former smokers (individuals who had smoked at least 100 cigarettes but were not currently smoking), and current smokers (those actively smoking with a lifetime total of at least 100 cigarettes).^[19]^ Alcohol consumption was classified into current drinkers (≥12 alcoholic beverages per year) and nondrinkers.^[20]^ Hypertension was defined through self-reported physician diagnosis. Participants reporting 2 or more clinical diagnoses of hypertension were categorized as hypertensive; all others were considered nonhypertensive.^[21]^ Physical activity levels were categorized as sedentary, moderate, or vigorous, based on participants’ self-reported engagement in at least 10-minute bouts during the preceding 30 days: moderate activity was defined as light sweating or mild-to-moderate increases in breathing or heart rate, whereas vigorous activity involved heavy sweating or substantial increases in exertion.^[21]^ Based on self-reported sleep duration, participants were categorized into 1 of 3 groups: insufficient sleep (<7 hours per day), recommended sleep (7–9 hours per day), or excessive sleep (>9 hours per day).^[22]^

### 2.6. Statistical analysis

All analyses incorporated sampling weights to generate estimates representative of the noninstitutionalized US population. For the fasting subsample, the provided 2-year MEC examination weights (WTSAF2YR) were used as the basis. The final sampling weight was calculated as WTSAF2YR divided by 4. In the descriptive analyses, categorical variables were summarized as unweighted counts (n) and weighted percentages. Continuous variables following a normal distribution were expressed as means ± standard deviations, whereas those with a skewed distribution were reported as medians with interquartile ranges. Group comparisons for continuous variables were conducted using analysis of variance for normally distributed data and the Kruskal–Wallis test for non‑normally distributed data. Categorical variables were compared via weighted chi‑square tests. Prior to regression modeling, multicollinearity among covariates was assessed with the variance inflation factor; a threshold of <5 was considered acceptable. Missing covariate data were addressed using complete‑case analysis. Given the cross‑sectional design and publicly available nature of the NHANES data, a prospective power calculation was not applicable.

Logistic regression models were employed to estimate odds ratios (ORs) and 95% confidence intervals (CIs) for the relationship between the TyG–RFM index (per 10-unit increment) and OSA risk. The exposure variable was analyzed in both continuous form and according to quintile groupings, with the lowest quintile serving as the reference category. Three multivariable models were constructed with sequential adjustment: model 1 included age, sex, and race/ethnicity; model 2 further adjusted for socioeconomic and lifestyle factors (education, marital status, poverty income ratio, smoking, alcohol consumption, BMI, physical activity, and sleep duration); and model 3 (fully adjusted) further adjusted for clinical comorbidities (hypertension, coronary heart disease, diabetes).

A restricted cubic spline model with 3 knots (placed at the 10th, 50th, and 90th percentiles) was fitted to evaluate potential nonlinear associations between the TyG–RFM index and OSA incidence. This model was adjusted for the full set of covariates from the fully adjusted model (demographic, socioeconomic, lifestyle, and clinical factors). Subgroup analyses were conducted to assess effect modification by prespecified factors: age (<60 vs ≥60 years), sex, race/ethnicity, BMI category (<25 vs ≥25 kg/m^2^), smoking status, alcohol consumption, hypertension, diabetes, and coronary heart disease. The significance of interaction terms was evaluated using likelihood ratio tests.

To assess the robustness of the primary findings, we conducted 2 sensitivity analyses. First, multivariable analyses were repeated using multiple imputation by chained equations to handle missing covariates. Second, the primary analyses were repeated after excluding participants aged older than 70 years to mitigate potential residual confounding from extreme age or survival bias.

All statistical analyses were conducted using R (version 4.3.1; R Foundation for Statistical Computing) and Free Statistics software (version 2.1.1; Beijing Free Clinical Medical Technology Co., Ltd.). Statistical significance was defined as a two‑sided *P* < .05.

## 3. Results

### 3.1. Participant characteristics

A total of 7283 participants were included in this study, with an overall mean age of 46.90 ± 16.7 years. Among them, 48.7% (weighted) were male (Table 1). Compared with participants in the lower tertiles (T1 and T2), those in the highest tertile (T3) were older, had a lower proportion of males, and exhibited a higher BMI. The prevalence of hypertension, diabetes, and OSA increased progressively across ascending tertiles of TyG–RFM. Specifically, the proportion of participants with OSA was 43.9% in T1, 49.9% in T2, and 54.2% in T3 (*P* < .001). Similarly, higher TyG–RFM was associated with lower physical activity levels, lower alcohol consumption, and lower household income. Significant differences were also observed across tertiles in terms of education level and marital status.

**Table 1:** Baseline characteristics across TyG–RFM tertiles.

Variables	Tertiles of TyG–RFM index				*P*
	Overall	T1 (71.5–267.6)	T2 (267.6–346.4)	T3 (346.4–600.0)	
Participants, n	7283	2428	2427	2328	
Age (yr), mean (SD)	46.90 (16.7)	41.9 (16.2)	48.6 (16.0)	50.9 (16.5)	<.001
Sex, male, n (%)	3586 (48.7)	2108 (83.2)	1312 (50.1)	166 (7.3)	<.001
Race/ethnicity, n (%)					<.001
Non-Hispanic White	3182 (68.9)	1041 (67.7)	1134 (71.2)	1007 (67.6)	
Non-Hispanic Black	1470 (10.5)	573 (11.8)	413 (8.4)	484 (11.2)	
Mexican American	1219 (8.2)	319 (7.4)	410 (8.0)	490 (9.3)	
Other race/ethnicity	1412 (12.5)	495 (13.1)	470 (12.5)	447 (11.8)	
Education level, n (%)					<.001
Less than high school	707 (4.8)	181 (4.0)	231 (4.3)	295 (6.5)	
High school or equivalent	2740 (34.6)	901 (32.9)	876 (33.0)	963 (38.6)	
College or above	3836 (60.5)	1346 (63.2)	1320 (62.7)	1170 (54.9)	
Marital status, n (%)					<.001
Never married	1184 (17.0)	572 (25.3)	317 (12.7)	295 (12.1)	
Married or living with a partner	4514 (64.8)	1513 (62.9)	1592 (68.9)	1409 (62.4)	
Widowed, divorced or separated	1585 (18.2)	343 (11.8)	518 (18.4)	724 (25.4)	
Poverty income ratio, median (IQR)	3.1 (1.7–5.0)	3.3 (1.7–5.0)	3.4 (1.8–5.0)	2.5 (1.4–4.4)	<.001
Body mass index (kg/m^2^), mean (SD)	29.1 (6.8)	24.8 (3.8)	28.9 (5.7)	34.3 (7.2)	<.001
Smoking status, n (%)					<.001
Never	3927 (53.2)	1197 (50.8)	1269 (53.2)	1461 (55.9)	
Former	1898 (26.2)	616 (24.5)	710 (28.3)	572 (25.8)	
Current	1458 (20.5)	615 (24.6)	448 (18.4)	395 (18.2)	
Alcohol consumption, n (%)	5487 (80.2)	2032 (86.4)	1920 (83.4)	1535 (69.5)	<.001
Hypertension, n (%)	2165 (25.4)	450 (14.4)	756 (26.7)	959 (36.7)	<.001
Coronary heart disease, n (%)	305 (3.5)	84 (2.6)	143 (4.9)	78 (2.8)	.001
Diabetes, n (%)	976 (9.2)	147 (3.6)	327 (8.5)	502 (16.7)	<.001
Physical activity, n (%)					<.001
Sedentary	3554 (41.6)	958 (33.1)	1194 (40.7)	1402 (52.5)	
Moderate	1917 (28.5)	545 (22.8)	671 (32.1)	701 (31.2)	
Vigorous	1812 (29.8)	925 (44.0)	562 (27.2)	325 (16.4)	
Sleep duration, n (%)					.001
<7 h	2300 (29.0)	769 (28.6)	817 (30.9)	714 (27.4)	
7–9 h	4502 (65.2)	1517 (66.7)	1464 (63.5)	1521 (65.3)	
>9 h	481 (5.8)	142 (4.7)	146 (5.6)	193 (7.4)	
Obstructive sleep apnea, n (%)	3616 (49.1)	1111 (43.9)	1219 (49.9)	1286 (54.2)	<.001

IQR = interquartile range, SD = standard deviation, T = tertile, TyG–RFM = triglyceride glucose–relative fat mass.

### 3.2. Association between TyG–RFM and OSA

Table 2 presents the results of multivariate logistic regression analyses examining the association between the TyG–RFM index and OSA. In the unadjusted model, each 10-unit increase in the TyG–RFM index was associated with a 3% increase in the odds of OSA (OR = 1.03; 95% CI, 1.02–1.04). After full adjustment for covariates in model 3, the risk of OSA increased by 4% per 10-unit increment (OR = 1.04; 95% CI, 1.02–1.06). When analyzed by quintiles of the TyG–RFM index, participants in the highest quintile (Q5) had a 113% higher risk of OSA compared with those in the lowest quintile (Q1) in the unadjusted model (OR = 2.13; 95% CI, 1.73–2.62). This association remained statistically significant after full adjustment in model 3 (OR = 1.95; 95% CI, 1.29–2.95). Figure 2 displays the fully adjusted restricted cubic spline curve illustrating the dose–response relationship between the TyG–RFM index and the odds of OSA. After adjusting for all covariates, the TyG–RFM index showed a generally linear positive association with OSA risk across its observed range (*P* for nonlinearity = .292). The OR progressively increased with higher TyG–RFM values.

**Table 2:** Association of TyG–RFM and obstructive sleep apnea.

Variables	n	Crude OR (95% CI)	*P*	Model 1 OR (95% CI)	*P*	Model 2 OR (95% CI)	*P*	Model 3 OR (95% CI)	*P*
TyG–RFM index (per 10-unit increment)	7283	1.03 (1.02–1.04)	<.001	1.09 (1.08–1.11)	<.001	1.04 (1.02–1.06)	<.001	1.04 (1.02–1.06)	<.001
TyG–RFM index quintiles									
Q1 (71.5–240.2)	1457	Reference		Reference		Reference		Reference	
Q2 (240.3–281.1)	1456	1.60 (1.32–1.95)	<.001	2.12 (1.73–2.61)	<.001	1.49 (1.18–1.89)	.001	1.49 (1.18–1.88)	.001
Q3 (281.2–327.8)	1457	1.48 (1.21–1.81)	<.001	2.66 (2.06–3.45)	<.001	1.41 (1.05–1.91)	.024	1.42 (1.05–1.90)	.022
Q4 (327.8–385.7)	1456	1.38 (1.12–1.68)	.002	3.90 (2.98–5.11)	<.001	1.55 (1.07–2.24)	.022	1.53 (1.06–2.21)	.023
Q5 (385.7–600.0)	1457	2.13 (1.73–2.62)	<.001	7.56 (5.64–10.13)	<.001	1.98 (1.30–3.02)	.002	1.95 (1.29–2.95)	.002
*P* for trend			<.001		<.001		.011		.012

Crude: unadjusted.

Model 1: adjusted for age, sex, and race/ethnicity.

Model 2: built upon model 1 with additional adjustment for socioeconomic and lifestyle factors (education, marital status, poverty income ratio, smoking, alcohol consumption, body mass index, physical activity, and sleep duration).

Model 3: further adjusted for comorbidities (hypertension, coronary heart disease, and diabetes).

CI = confidence interval, OR = odds ratio, Q = quintile, TyG–RFM = triglyceride glucose–relative fat mass.

**Figure 2.**
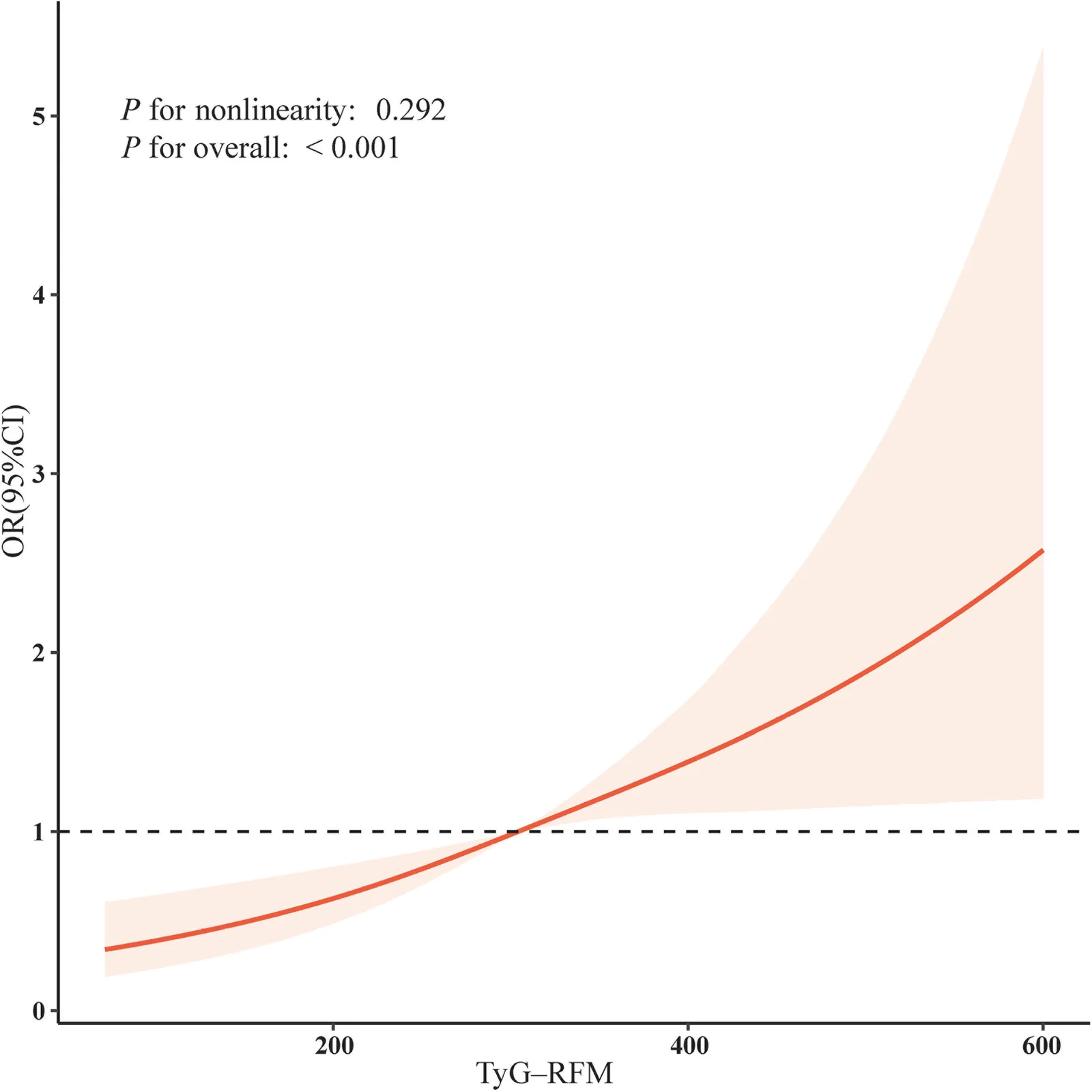
Association between TyG–RFM and the odds ratio of obstructive sleep apnea. The solid red line denotes the odds ratio, with the surrounding pink band indicating its 95% CI, as per the covariate adjustment specifications of model 3. CI = confidence interval, OR = odds ratio, TyG–RFM = triglyceride glucose–relative fat mass.

### 3.3. Subgroup analyses

To validate the stability of the association between the TyG–RFM index and OSA risk, a subgroup analysis was performed (Fig. 3). No significant interaction was observed across subgroups stratified by age, sex, race/ethnicity, BMI, smoking status, alcohol consumption, hypertension, coronary heart disease, or diabetes (all *P* for interaction > .05). Nevertheless, the association appeared stronger in males, non-Hispanic Black individuals, Mexican Americans, other racial/ethnic groups, and participants with hypertension or coronary heart disease. In contrast, an inverse association was suggested in individuals with BMI <25 kg/m^2^ and those with diabetes. Of note, the *P* values for interaction approached significance for hypertension (*P* = .051) and coronary heart disease (*P* = .083).

**Figure 3.**
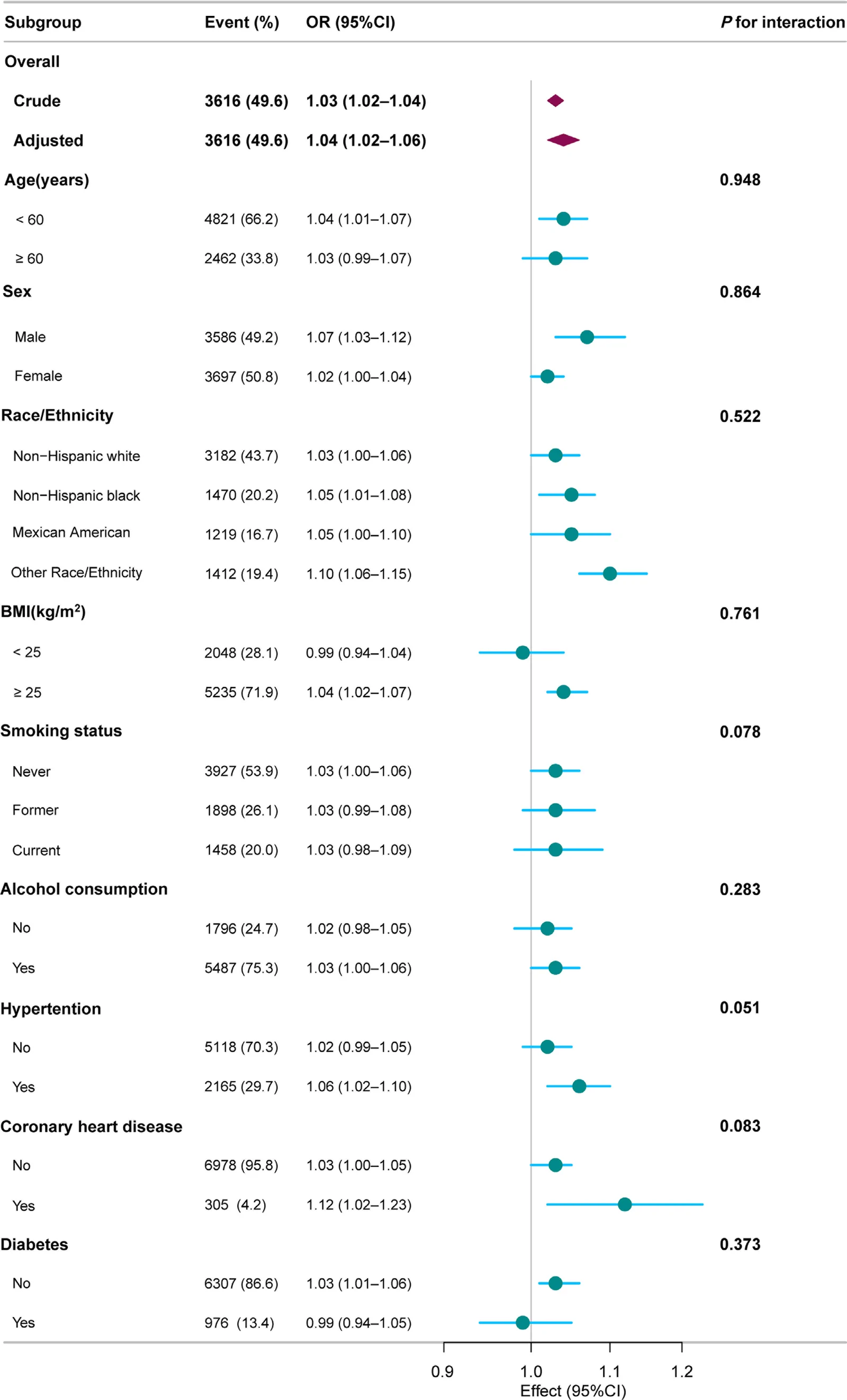
Subgroup analyses of TyG–RFM and obstructive sleep apnea. The ORs and their 95% CIs are indicated by green dots and horizontal blue lines, respectively; summary estimates are shown as diamonds, whose center lines and widths correspond to the odds ratios and confidence intervals. These results were produced using the covariate adjustments specified in model 3, excluding the stratification variable itself. BMI = body mass index, CI = confidence interval, OR = odds ratio.

### 3.4. Sensitivity analysis

To assess the stability of the relationship between the TyG–RFM index and OSA risk, we performed 2 sensitivity analyses. After multiple imputation of covariates (Table 3, unweighted n = 8462), the OR for TyG–RFM per 10-unit increase was 1.04 (95% CI, 1.03–1.06). Similarly, after excluding participants older than 70 years (Table 4, unweighted n = 6210), the corresponding OR was 1.04 (95% CI, 1.02–1.06). Both sensitivity analyses yielded effect estimates nearly identical to the primary result (OR = 1.04; 95% CI, 1.02–1.06), confirming the stability of the association.

**Table 3:** Sensitivity analysis following the multiple imputation of covariates.

Variables	n	Crude OR (95% CI)	*P*	Model 4 OR (95% CI)	*P*
TyG–RFM index (per 10-unit increment)	8462	1.03 (1.02–1.03)	<.001	1.04 (1.03–1.06)	<.001
TyG–RFM index quintiles					
Q1 (71.5–240.7)	1693	Reference		Reference	
Q2 (240.7–282.1)	1692	1.64 (1.37–1.96)	<.001	1.55 (1.25–1.93)	<.001
Q3 (282.1–329.7)	1692	1.51 (1.27–1.80)	<.001	1.50 (1.16–1.94)	.003
Q4 (329.7–387.0)	1692	1.43 (1.18–1.74)	<.001	1.70 (1.21–2.41)	.004
Q5 (387.0–600.0)	1693	2.07 (1.72–2.51)	<.001	2.08 (1.39–3.11)	<.001
*P* for trend			<.001		.002

Crude: unadjusted.

Model 4: covariate adjustment matched model 3 specifications.

CI = confidence interval, OR = odds ratio, Q = quintile, TyG–RFM = triglyceride glucose–relative fat mass.

**Table 4:** Sensitivity analysis in participants aged ≤70 years.

Variables	n	Crude OR (95% CI)	*P*	Model 5 OR (95% CI)	*P*
TyG–RFM index (per 10-unit increment)	6210	1.03 (1.02–1.04)	<.001	1.04 (1.02–1.06)	<.001
TyG–RFM index quintiles					
Q1 (71.5–238.0)	1242	Reference		Reference	
Q2 (238.1–278.2)	1242	1.62 (1.32–1.99)	<.001	1.51 (1.17–1.94)	.002
Q3 (278.3–325.1)	1242	1.62 (1.31–1.99)	<.001	1.58 (1.15–2.16)	.005
Q4 (325.2–384.2)	1242	1.52 (1.22–1.90)	<.001	1.70 (1.15–2.51)	.009
Q5 (384.2–600.0)	1242	2.40 (1.91–3.01)	<.001	2.22 (1.40–3.53)	.001
*P* for trend			<.001		.004

Crude: unadjusted.

Model 5: covariate adjustment matched model 3 specifications.

CI = confidence interval, OR = odds ratio, Q = quintile, TyG–RFM = triglyceride glucose–relative fat mass.

## 4. Discussion

This cross-sectional study indicated a linear positive association between the TyG–RFM index and OSA risk among US adults. Stratified and sensitivity analyses confirmed the robustness of this association.

Previous studies have established the TyG index as a direct surrogate marker of insulin resistance and linked it to the risk of OSA,^[23,24]^ which aligns with our findings. In parallel, the RFM index has been validated as a measure that estimates whole-body fat percentage more accurately than BMI.^[25]^ Consistent with our results, Guo et al^[12]^ reported a positive correlation between RFM and OSA. Notably, recent research combining TyG with adiposity indices (e.g., TyG–BMI, TyG–WC) suggests that such composite metrics may offer superior predictive value for conditions including nonalcoholic fatty liver disease and OSA risk compared with single parameters.^[26,27]^ Building on this integrative concept, the present study synthesized TyG (reflecting metabolic status) and RFM (reflecting adiposity) into a novel composite index. By directly investigating its association with OSA risk, our work extends previous findings and specifically explores the interplay between systemic metabolism, adiposity, and OSA pathogenesis.

The observed association between the TyG–RFM ratio and OSA may be underpinned by several biologically plausible mechanisms. An elevated TyG index reflects insulin resistance and atherogenic dyslipidemia, both of which are implicated in systemic inflammation, endothelial dysfunction, and alterations in the neuromuscular control of the upper airway.^[28,29]^ Concurrently, an increased RFM signifies central obesity with visceral fat accumulation. Fat deposition in the neck and peripharyngeal area can mechanically compromise the upper airway, predisposing individuals to OSA.^[30]^ Furthermore, OSA itself, characterized by intermittent hypoxia and sleep fragmentation, can exacerbate insulin resistance, dyslipidemia, and obesity via sympathetic activation, oxidative stress, and inflammatory pathways, creating a bidirectional vicious cycle.^[31]^ Thus, our composite index, integrating both metabolic and adiposity signals, plausibly captures these convergent pathophysiological drivers that underlie OSA susceptibility and its systemic sequelae.

This study is subject to several limitations. First, due to its cross-sectional nature, the observed association between the TyG–RFM index and OSA cannot support causal interpretation; prospective longitudinal studies are needed to establish temporality and confirm the relationship. Second, OSA was defined based on questionnaire data in NHANES rather than by the gold-standard polysomnography, which may lead to outcome misclassification and potential under- or overestimation of the true disease prevalence. Third, despite adjustment for a comprehensive set of covariates, residual confounding from unmeasured factors – such as neck circumference, waist-to-hip ratio, and continuous positive airway pressure therapy – may still be present. Finally, while the use of NHANES data enhances the generalizability of our findings to the noninstitutionalized US adult population, the results may not be directly applicable to other ethnic or geographic groups.

## 5. Conclusions

This cross-sectional study showed a positive linear association between the TyG–RFM index and the risk of OSA among US adults, where a higher TyG–RFM index was associated with a greater likelihood of OSA. These findings suggest that the TyG–RFM index may serve as a practical tool for OSA risk stratification and early screening. Prospective studies are needed to confirm these observational results.

## Acknowledgments

We extend our sincere appreciation to the National Center for Health Statistics (NCHS) and all National Health and Nutrition Examination Survey (NHANES) participants, whose contributions were essential to the conduct of this research.

## Author contributions

**Conceptualization:** Yunzhou Zheng, Mingqiang Mi.

**Data curation:** Yunzhou Zheng, Shanshan Zhang.

**Formal analysis:** Yunzhou Zheng, Mingqiang Mi.

**Funding acquisition:** Yunzhou Zheng.

**Methodology:** Yunzhou Zheng, Mingqiang Mi.

**Supervision:** Yunzhou Zheng.

**Validation:** Yunzhou Zheng, Shanshan Zhang.

**Writing – original draft:** Yunzhou Zheng, Mingqiang Mi.

**Writing – review & editing:** Yunzhou Zheng, Shanshan Zhang.

**Investigation:** Shanshan Zhang.

**Project administration:** Shanshan Zhang.

**Resources:** Shanshan Zhang.

**Visualization:** Mingqiang Mi.
